# Unchanged right ventricular strain in repaired tetralogy of Fallot after pulmonary valve replacement with radial long-axis cine magnetic resonance images

**DOI:** 10.1038/s41598-021-98464-0

**Published:** 2021-09-23

**Authors:** Masateru Kawakubo, Yuzo Yamasaki, Daisuke Toyomura, Kenichiro Yamamura, Ichiro Sakamoto, Tetsuhiro Moriyama, Hidetake Yabuuchi, Kousei Ishigami

**Affiliations:** 1 Department of Health Sciences, Faculty of Medical Sciences, Kyushu University, 3-1-1 Maidashi, Higashi-ku, Fukuoka-shi, Fukuoka, 812-8582 Japan; 2 Department of Clinical Radiology, Graduate School of Medical Sciences, Kyushu University, Fukuoka, Japan; 3 Department of Pediatrics, Graduate School of Medical Sciences, Kyushu University, Fukuoka, Japan; 4 Department of Cardiovascular Medicine, Kyushu University Hospital, Fukuoka, Japan; 5 Institute of Mathematics for Industry, Kyushu University, Fukuoka, Japan

**Keywords:** Congenital heart defects, Magnetic resonance imaging, Three-dimensional imaging

## Abstract

We measured right ventricular (RV) strain by applying a novel postprocessing technique to conventional short-axis cine magnetic resonance imaging in the repaired tetralogy of Fallot (TOF) and investigated whether pulmonary valve replacement (PVR) changes the RV strain. Twenty-four patients with repaired TOF who underwent PVR and 16 healthy controls were enrolled. Global maximum and minimum principal strains (GPS_max_, GPS_min_) and global circumferential and longitudinal strains (GCS, GLS) were measured from short-axis cine images reconstructed radially along the long axis. Strain parameters before and after PVR were compared using paired t-tests. One-way ANOVA with Tukey post-hoc analysis was used for comparisons between the before and after PVR groups and the control group. There were no differences in strain parameters before and after PVR. The GPS_max_ before PVR was lower than that in the control group (*P* = 0.002). Before and after PVR, GCSs were higher and GLSs were lower than those in the control group (before and after GCSs: *P* = 0.002 for both, before and after GLSs: *P* < 0.0001 and *P* = 0.0003). RV strains from radially reconstructed short-axis cine images revealed unchanged myocardial motion after PVR. When compared to the control group, changes in GCS and GLS in TOF patients before and after PVR might be due to RV remodeling.

## Introduction

Patients with surgically repaired TOF experience significant morbidity and mortality related to biventricular dysfunction and arrhythmia in their adult years^1–3^. These sequelae are believed to be in part related to chronic pulmonary regurgitation caused by efforts to relieve pulmonary valve stenosis with the initial repair. PVR is performed to improve pulmonary regurgitation or valvular stenosis and improve long-term outcomes^4,5^ in adult patients with repaired TOF. However, cardiovascular magnetic resonance (CMR) feature tracking showed that ventricular strain was unchanged, even after PVR^6^.

Peak strain is a helpful marker for quantifying myocardial tissue deformation^7–9^, especially in the RV, because the image quality of echocardiography is sometimes inadequate^10,11^. Generally, the RV strain is evaluated using circumferential strain (CS) or longitudinal strain (LS) from the motion of the endocardium in one plane of the ventricular short- or long-axis, respectively. However, the motion of the heart is three-dimensional. Accordingly, such predefined directions of change may not reliably describe deformation in the dominant direction of tissue movement established by engaged myocardial fibers. On the other hand, three-dimensional image acquisition for myocardial strain is technically difficult and time-consuming, if possible. Recently, the feasibility of three-dimensional image analysis using routine cine CMR images for the estimation of principal strain (PS), a geometry-independent measure established from the dominant direction of local tissue deformation, has been reported^12^. Therefore, we measured RV PS of three-dimensional “real” strain in repaired TOF by applying this novel postprocessing technique to conventional two-dimensional cine imaging. In the present study, we investigated whether PVR changes the RV PS. We also investigated the clinical impact of the relationship between strain parameters and clinical and hemodynamic measurements.

## Results

Table 1 shows the baseline characteristics, CMR RV parameters, and cardiac catheterization parameters. After PVR, the World Health Organization class and RV volume parameters showed significant improvement.

**Table 1:** Baseline clinical characteristics, CMR right ventricular parameters, and right heart catheterization parameters.

	TOF patients (n = 24)		*P-*value	Control (n = 16)
	Before PVR	After PVR		
Age before PVR (y)	26 ± 9			33 ± 11
Male/female	11/13			13/3
Body surface area before PVR (m^2^)	1.58 ± 0.17			1.76 ± 0.13
WHO classification (I/II/III/IV)	17/5/2/0	23/1/0/0		16/0/0/0
CMR to PVR duration (day)	252 ± 158			
PVR to CMR duration (day)		433 ± 163		
**CMR RV parameters**				
HR (bpm)	70 ± 12	69 ± 9	0.41	66 ± 14
RVEDVi (mL/m^2^)	165.7 ± 41.9	108.7 ± 19.0	** < 0.0001**	71.0 ± 18.0
RVESVi (mL/m^2^)	94.2 ± 29.7	66.0 ± 14.7	** < 0.0001**	28.8 ± 10.9
RVEF (%)	43.8 ± 7.8	39.3 ± 7.1	**0.02**	60.5 ± 6.3
RVSVi (mL/m^2^)	71.4 ± 19.5	42.7 ± 10.3	** < 0.0001**	42.2 ± 8.1
**Cardiac catheterization**				
RVP (mmHg)	51.9 ± 18.8			
LVP (mmHg)	122.3 ± 17.0			
PA pressure (mmHg)	34.5 ± 12.0			
RVP/ LVP	0.43 ± 0.18			
RV-PA pressure gradient	17.4 ± 17.3			

*CMR* cardiovascular magnetic resonance, *EDVi* indexed end-diastolic volume, *EF* ejection fraction, *ESVi* indexed end-systolic volume, *HR* heart rate, *LVP* left ventricular pressure, *PA* pulmonary artery, *PVR* pulmonary valve replacement, *RV* right ventricular, *RVP* right ventricular pressure, *SVi* stroke volume index, *TOF* tetralogy of Fallot, *WHO* World Health Organization. Significantly differences are expressed as bold *P*-values.

**Figure 1:**
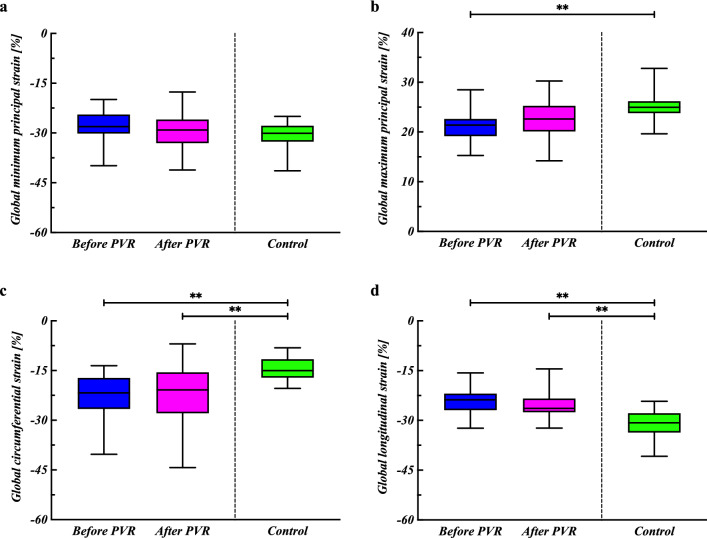
Global strain values of patients with tetralogy of Fallot before and after pulmonary valve replacement, and of control patients. The (**a**) global minimum principal strains, (**b**) global maximum principal strains, (**c**) global circumferential strains, and (**d**) global longitudinal strains are shown as box-and-whisker plots. **P < 0.01, *P < 0.05.

**Figure 2:**
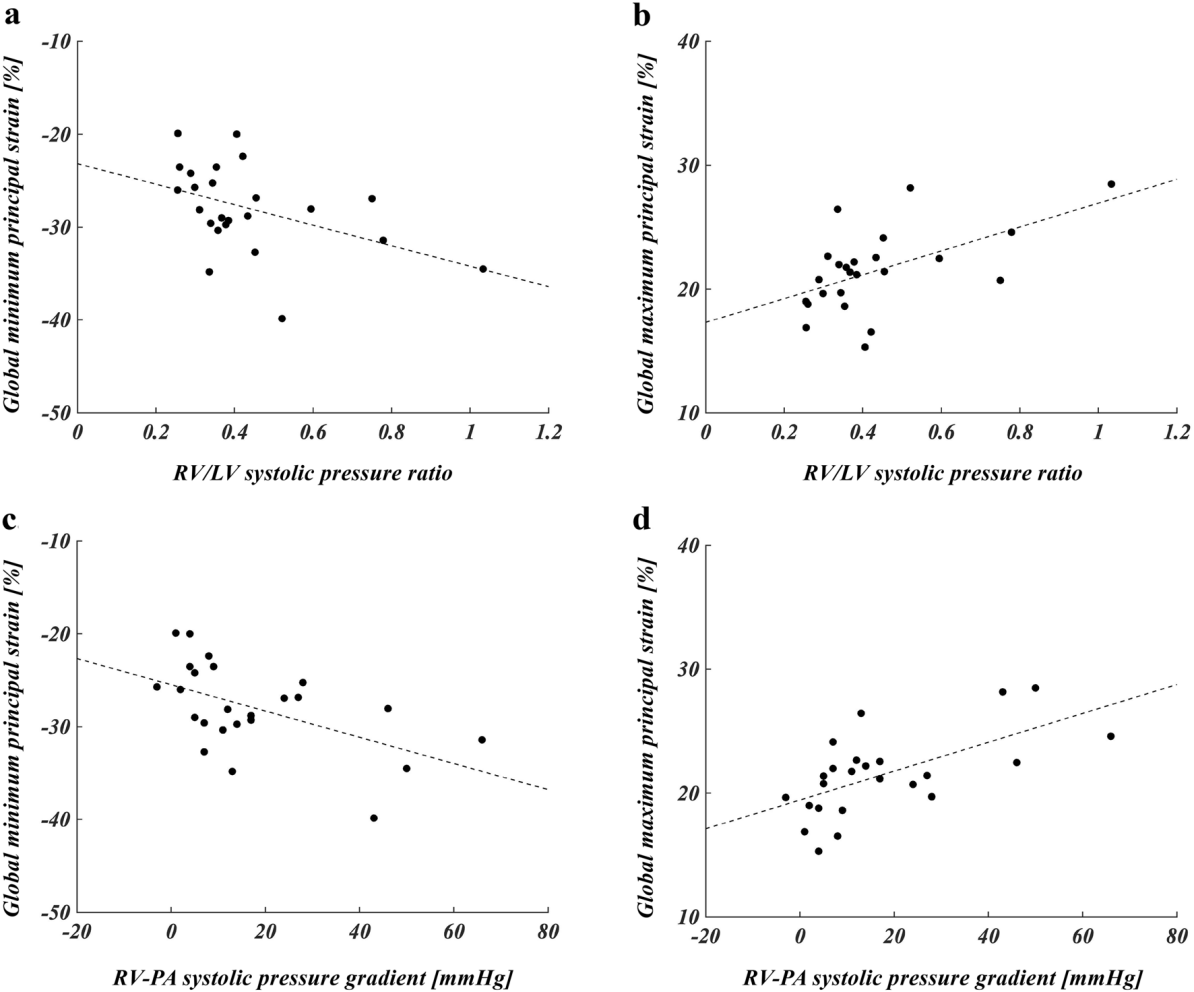
Correlations between global principal strains and blood pressure parameters derived from right heart catheterization. Scatter plots show the correlation of the systolic right to left ventricle (RV/LV) pressure ratio to (**a**) global minimum principal strain and (**b**) global maximum principal strain. Scatter plots show the correlation of the systolic right ventricular pulmonary artery (RV-PA) pressure gradient to the (**c**) global minimum principal strain and (**d**) global maximum principal strain.

Figure 1 shows the global strain parameters in the patients before and after PVR and in the control group. There were no differences in any of the strain parameters before and after PVR. The GPS_max_ before PVR was lower than that in the control group (*P* = 0.002). GCSs before and after PVR were higher than those in the control groups (*P* = 0.002 for both). GLSs before and after PVR were lower than those in the controls (*P* < 0.0001 and *P* = 0.0003). The *r*_*S*_ (confidence intervals) indicated significant correlations between the GPS_min_ and RVP/ LVP (*r*_*S*_ = − 0.44 [− 0.72, − 0.03]) and between the GPS_min_ and the RV-PA pressure gradient (*r*_*S*_ = − 0.54 [− 0.78, − 0.16]) (Fig. 2). In addition, the GPS_max_ was significantly correlated with RVP/ LVP and with the RV-PA pressure gradient (*r*_*S*_ = 0.47 [0.08, 0.74], *r*_*S*_ = 0.64 [0.31, 0.83]) (Fig. 2).

There were strong correlations between intra- and inter-observer strains (*P* < 0.01). Excellent intra-observer and inter-observer reproducibility were observed for RV strain measurements using radial long-axis reconstructed cine images (Table 2).

**Table 2:** Reproducibility of strain measurements.

	Intra-observer reproducibility				Inter-observer reproducibility			
Strain parameters	*r* (95% CI)	Bias (LOA)	SDD	ICC (95% CI)	*r* (95% CI)	Bias (LOA)	SDD	ICC (95% CI)
GPS_min_	0.95 (0.81, 0.99)	− 0.7% (− 3.9, 2.5)	1.6	0.94 (0.78–0.98)	0.84 (0.45, 0.96)	− 0.1% (− 5.5, 5.4)	2.8	0.83 (0.46–0.96)
GPS_max_	0.94 (0.75, 0.99)	0.4% (− 2.1, 3.5)	1.4	0.92 (0.74–0.98)	0.85 (0.49, 0.96)	0.1% (− 3.9, 4.0)	2.0	0.85 (0.52–0.96)
GCS	0.80 (0.35, 0.95)	− 0.4% (− 9.8, 8.9)	4.8	0.82 (0.45–0.95)	0.86 (0.49, 0.97)	0.9% (− 7.5, 9.4)	4.3	0.86 (0.52–0.96)
GLS	0.89 (0.59, 0.97)	− 1.2% (− 6.2, 3.8)	2.6	0.88 (0.60 − 0.97)	0.86 (0.50, 0.97)	− 1.1% (− 7.0, 4.9)	3.0	0.86 (0.54–0.96)

*r* Pearson’s correlation coefficients, *GPS*_*min*_ global minimum principal strain, *GPS*_*max*_ global maximum principal strain, *GCS* global circumferential strain, *GLS* global longitudinal strain, *LOA* limit of agreement, *SDD* standard deviation of the difference, *ICC* intraclass correlation coefficient, *CI* confidence interval.

## Discussion

According to our investigation, there were no differences in global PS values before and after PVR in adult patients with surgically repaired TOF. Furthermore, GCS and GLS were unchanged before and after PVR. Our results are in line with those of previous research^6^. Therefore, PVR in patients with repaired TOF led to no changes in RV global PS. There were no differences in global PS between the patients after PVR and the control group, although the RV volume parameters were not normalized. This may indicate that RV myocardial motility is maintained by remodeling, corresponding to long-term pressure overloading. The mean RV ESVi in the patients before PVR was 94 mL/m^2^, which corresponds to the previously reported adaptive RV remodeling conditions (73 to 113 mL/m^2^)^13^. Furthermore, in RV pressure overload, it is mainly the middle myocardial layer that hypertrophies^14,15^. The RV in patients with adapted remodeling is similar to the normal left ventricle, which has a well-developed middle circumferential layer^16^. Thus, the increase in the circumferential fiber mass may also contribute to the predominant circumferential RV free-wall shortening. According to our results, increased GCS and decreased GLS in the TOF group relative to the control group were consistent with these reports. Furthermore, our results suggest that even if the RV volume is reduced by PVR, there is no reverse remodeling of myocardial motion 1 year after the surgery. Earlier surgical intervention might be preferable to prevent myocardial remodeling.

In clinical situations, pressure parameters derived from cardiac catheterization are important indicators for PVR. We found that CMR-derived GPS_min_ and GPS_max_ were significantly correlated. The advantage of CMR with respect to cardiac catheterization is its accurate volume measurement and low invasiveness. However, its weakness is an inability to measure the pressure in the cardiac cavity or blood vessels. Surgical indications for PVR include volumetric parameters (e.g., RV dilatation) and pressure parameters (e.g., RVP/LVP). The estimation of pressure-derived parameters using our method has the potential to overcome CMR’s weakness. Because our strain analysis requires only short-axis cine CMR, a well-established method without the need for additional imaging, a wider range of applications could be expected.

We acknowledge that our strain analysis with radially reconstructed long-axis cine CMR was based on the original in-house algorithm. Although measurements of RV PS with echocardiography using commercial software have been reported^17,18^, no software has been established for RV PS for CMR. We considered that the values of PS, GCS, and GLS were clinically reasonable and reliable in the study populations. Furthermore, the reproducibility of the strain measurements with radially reconstructed long-axis images was compared with CMR-derived strain measurements using feature tracking^19,20^. However, a future validation study by comparing the commercially available software with echocardiography would be required. Additionally, it will be necessary to verify the technique by applying the analysis to different right heart diseases and investigating their relationship with other clinical indicators and prognoses.

In this cohort, the three-dimensional PS revealed unchanged RV myocardial motion after PVR. In TOF patients before and after PVR, changes in GCS and GLS with respect to the control group might be due to RV remodeling. PS values reconstructed by short-axis CMR images were correlated with the parameters of pressure overloading with cardiac catheterization.

## Methods

### Patients

This retrospective observational study was approved by the institutional review board of Kyushu University and conducted in accordance with the 1964 Declaration of Helsinki. Written informed consent was obtained from each patient. Twenty-four patients with surgically repaired TOF who underwent PVR were enrolled in the study, and datasets derived between October 2012 and March 2018 were analyzed. All patients were older than 18 years and underwent CMR assessment and cardiac catheterization of the right and left heart before PVR. In addition, CMR control data were obtained from 16 age- and sex-matched healthy subjects.

### Cardiac catheterization

All patients with repaired TOF underwent left and right heart catheterization before PVR. Cardiac catheterization provided pressure parameters of the left ventricular, right ventricular, and pulmonary arteries.

### Cine CMR imaging

Cine CMR was performed following a previous research^21^ using a 3.0T clinical scanner (Ingenia 3.0T CX; Philips Healthcare) with a dStream Torso coil using electrocardiographic gating. Steady-state free precession images were obtained using multi-breath holding with retrospective electrocardiogram gating in the axial and short-axis views covering the entire right ventricle with 20 phases per cardiac cycle. The axial cine imaging parameters were as follows: repetition time = 2.4 ms, echo time = 1.18 ms, SENSE factor = 2.0, flip angle = 35°, slice thickness = 8 mm, slice gap = 0 mm, field of view = 380 × 380 mm, acquisition matrix 176 × 245, and reconstruction matrix 448 × 448. The short-axis cine imaging parameters were as follows: repetition time = 2.7 ms, echo time = 1.37 ms, SENSE factor = 2.0, flip angle = 45°, slice thickness = 8 mm, slice gap = 0 mm, field of view = 380 × 435 mm, acquisition matrix 176 × 142, and reconstruction matrix 512 × 512. RV volume analysis was performed semi-automatically with subsequent manual correction using a workstation (IntelliSpace Portal, Philips Healthcare). RV volumes were measured using axial images, as previously reported^22^. End-diastolic volume (EDV) and end-systolic volume (ESV) were indexed to the body surface area. The ejection fraction (EF) was calculated using EDV and ESV. Stroke volume index (SVi) was calculated as the difference between indexed EDV and ESV values (EDVi and ESVi, respectively). The cardiac index was the product of the SVi and the heart rate (HR).

### Strain analysis with CMR

**Figure 3:**
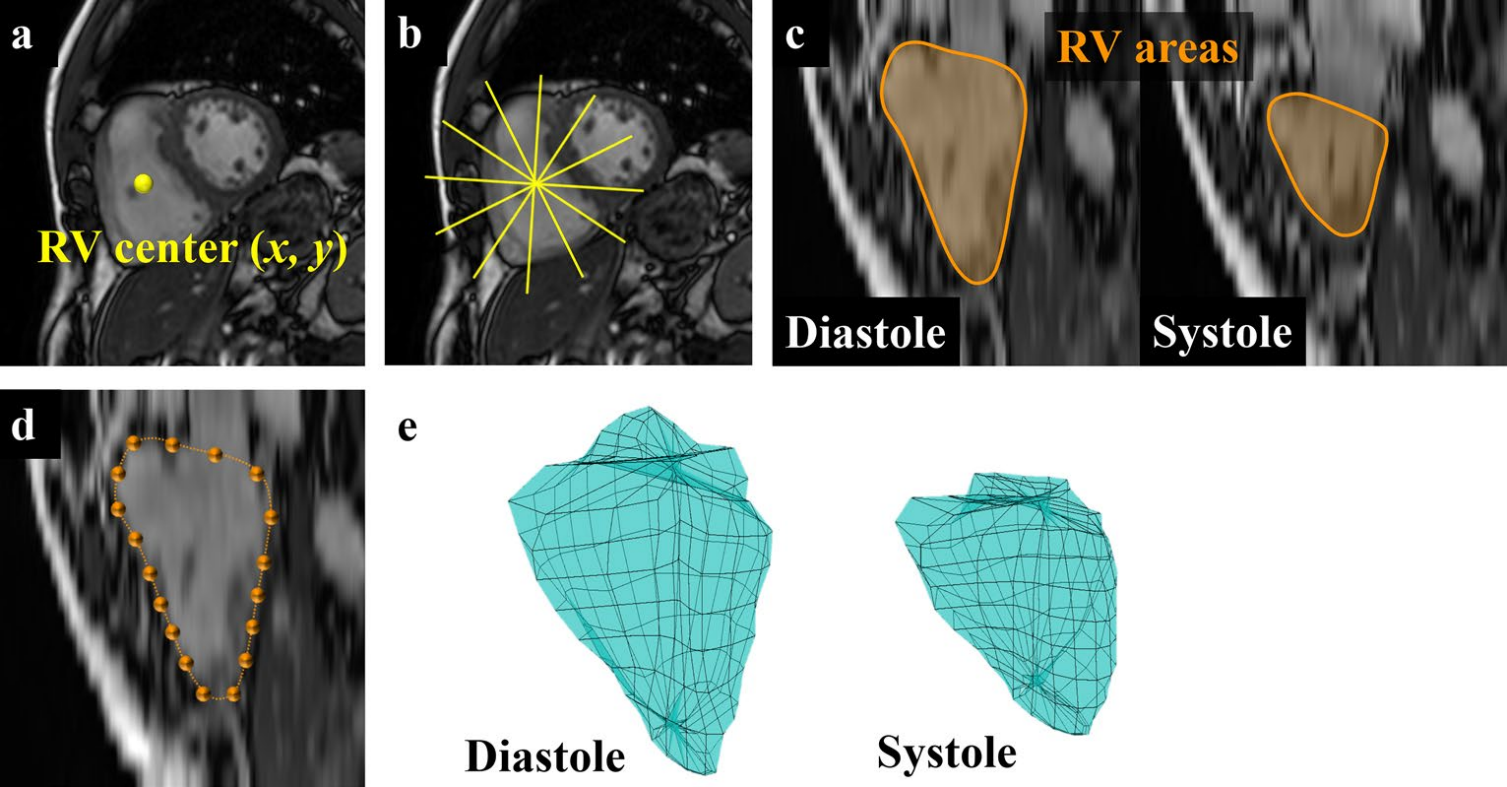
Segmentation of the right ventricle with radial long-axis reconstruction of short-axis cine magnetic resonance images. (**a**) The point () of right ventricular center is manually determined on the middle slice of the short-axis image in systole. (**b**) The images interpolated to the slice direction with the nearest-neighbor method are then automatically reconstructed into 6 radial long-axis images (). (**c**) Right ventricular areas are manually segmented on radially reconstructed images at the end-diastolic and end-systolic frames. (**d**) Endocardial lines are automatically replaced as evenly distributed points (). (**e**) Three-dimensional coordinates are automatically obtained at the end-diastolic and end-systolic frames. The right ventricular endocardial surface is individually modeled with these points in each patient.

**Figure 4:**
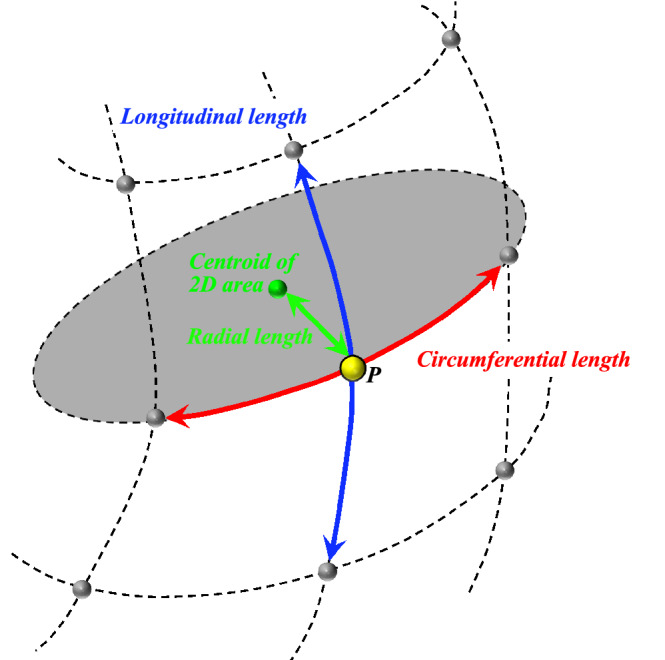
Strain calculations at the point of right ventricle. Regional strains at the point of right ventricular center () are determined as the percentage of the differences between the length at systole and diastole with respect to the length at diastole. The green point indicates the centroid of a two-dimensional area. The principal strain is calculated from three orthogonal regional strains.

All image processing algorithms for CMR-derived strain analyses were implemented using MATLAB R2020a (MATLAB Runtime Version 9.8; The MathWorks Inc.). Strain values were calculated from the three-dimensional coordinates of the RV endocardial point using standard short-axis cine CMR images. First, the point of the RV center was manually determined on the mid-heart short-axis slice during systole (Fig. 3a). The images interpolated to the slice direction using the nearest-neighbor method were then automatically reconstructed into six radial long-axis images at 30° around the RV point (Fig. 3b). Radial long-axis reconstructions were performed at the end-diastolic and end-systolic frames. Second, the RV areas were manually segmented on 12 images of six long-axis images with radial projection at the end-diastolic and end-systolic frames (Fig. 3c). Then, the RV endocardial lines were automatically replaced with evenly distributed points (Fig. 3d). Eighteen points were included in this study. Finally, the three-dimensional coordinates of 108 points of RV per frame (18 points for every 6 projections) were automatically obtained at the end-diastolic and end-systolic frames. The RV endocardial surface was individually modeled with these points for patient (Fig. 3e). Regional strains (*ε*), the percentages at each point, were calculated from the orthogonal lengths of circumferential, longitudinal, and radial directions at end-systole with respect to the lengths at end-diastole (Fig. 4) using Eq. (1):

1 $$\varepsilon \left[ \% \right] = \frac{{\left( {systolic\,length - diastolic\,length} \right)}}{diastolic\,length} \times 100$$

Further, the maximum and minimum PS were calculated from the three orthogonal regional strains, as per the previous research^12^. In the present study, the GPS_max_ and GPS_min_ were determined as the mean values of maximum and minimum PS of all RV endocardial points, respectively. The GCS and GLS were the means of the strains in the circumferential and longitudinal directions for all RV endocardial points, respectively.

### Statistical analysis

All statistical analyses were performed using GraphPad Prism version 8.1.2. (GraphPad Software, Inc.). Differences were considered statistically significant at *P* < 0.05. The Shapiro–Wilk test was used to evaluate the normality of the data distribution, and the mean and standard deviations were calculated. All strain parameters before and after PVR were compared using paired t-tests. One-way ANOVA with Tukey post-hoc analysis, was used for the comparisons between patient groups before and after PVR and the control group. Spearman correlation coefficients (*r*_*S*_) between the GPS_min_ and the right ventricular to left ventricular pressure (RVP/ LVP) ratio and between the GPS_min_ and RV to pulmonary artery (PA) pressure gradient were calculated. Similarly, the *r*_*S*_ for GPS_max_ were also calculated.

### Intra-observer and inter-observer reproducibility

The intra-observer reproducibility of the image analysis for strain calculations was evaluated following a previous research^23^. A single observer performed all analyses for 10 randomly selected patients and then blindly repeated the analyses at least 1 month later. Inter-observer reproducibility was evaluated based on the same 10 patients and performed by a second observer who was blinded to the clinical and experimental data. Pearson’s correlation coefficients of intra- and inter-observer strain parameters were calculated. The intra-observer and inter-observer reproducibility of the strain measurements was evaluated using Bland–Altman analyses and intraclass correlation coefficients (ICC) with one-way or two-way random single measures (ICC [1, 1] or ICC [1, 2], respectively). The ICC values were defined as excellent (≥ 0.75), good (0.60–0.74), moderate (0.40–0.59), or poor (≤ 0.39).

## Data Availability

Reprints and permissions information is available.
